# Modulation of mitochondrial ion transport by inorganic polyphosphate - essential role in mitochondrial permeability transition pore

**DOI:** 10.1007/s10863-016-9650-3

**Published:** 2016-02-18

**Authors:** Artyom Y. Baev, Alexander Negoda, Andrey Y. Abramov

**Affiliations:** 1 National University of Uzbekistan, Tashkent, Uzbekistan; 2 Department of Physiology and Biophysics, Dalhousie University, Halifax, Canada; 3 Department of Molecular Neuroscience, UCL Institute of Neurology, Queen Square, London, WC1N 3BG UK

**Keywords:** Inorganic polyphosphate, Mitochondrial permeability transition pore, Ca^2+^

## Abstract

Inorganic polyphosphate (polyP) is a biopolymer of phosphoanhydride-linked orthophosphate residues. PolyP is involved in multiple cellular processes including mitochondrial metabolism and cell death. We used artificial membranes and isolated mitochondria to investigate the role of the polyP in mitochondrial ion transport and in activation of PTP. Here, we found that polyP can modify ion permeability of de-energised mitochondrial membranes but not artificial membranes. This permeability was selective for Ba^2+^ and Ca^2+^ but not for other monovalent and bivalent cations and can be blocked by inhibitors of the permeability transition pore – cyclosporine A or ADP. Lower concentrations of polyP modulate calcium dependent permeability transition pore opening. Increase in polyP concentrations and elongation chain length of the polymer causes calcium independent swelling in energized conditions. Physiologically relevant concentrations of inorganic polyP can regulate calcium dependent as well calcium independent mitochondrial permeability transition pore opening. This raises the possibility that cytoplasmic polyP can be an important contributor towards regulation of the cell death.

## Introduction

Inorganic polyphosphates (polyP) are the polymers that consist of many orthophosphate residues linked together via phosphoanhydride bonds similar of those of ATP. PolyP was found in all living organisms tested, but its concentration and size vary significantly depending on type of the organism, specific tissue and cellular localization. In microorganisms polyP is involved in a number of diverse processes including gene transcription, energy and inorganic phosphate storage and metal chelation (Kulakovskaya and Kulaev, 2013; Rao et al., 2009). In mammalian cells polyP plays a role in blood coagulation, enzymatic activity, cell death, and as a signaling molecule in central nervous system (Gray et al., 2014; Holmstrom et al., 2013; Morrissey et al., 2012). Disbalance in polyP homeostasis has been found in number of cell disease models including models of Parkinson’s disease (Angelova et al., 2014).

PolyP likely plays an integral role in mitochondrial energy metabolism. Indeed, enzymatic depletion of polyP in mitochondria significantly alters mitochondrial function, while activation of mitochondrial respiration induces polyP production by this organelle (Abramov et al., 2007; Pavlov et al., 2010). One of the intriguing aspects of polyP functioning is its possible involvement in the mitochondrial Permeability Transition Pore (PTP). In 1988 Reusch and Sadoff demonstrated that competent *E. Coli* contain an ion channel formed by polyP, poly-β-hydroxybutyrate (PHB) and Ca^2+^ ions which was selective for cations and especially for calcium (Reusch and Sadoff, 1988). Later, the similar channel-forming complex of polyP/PHB/Ca^2+^ was purified from the mammalian mitochondria (Pavlov et al., 2005). Interestingly, when isolated from mitochondria and reconstituted into planar lipid bilayers (BLM) polyP/PHB/Ca^2+^ complex showed properties similar to PTP (Pavlov et al., 2005). Reduction of mitochondrial polyP concentration in mitochondria, via expression of the mitochondrially targeted exopolyphosphatase from yeast, increased calcium capacity of mitochondria, decreased probability of PTP opening and protected against calcium induced cell death (Abramov et al., 2007). The possible role of the polyP in activation of PTP was also confirmed in cardiac cells (Seidlmayer et al., 2012). However, the mechanism of the polyP contribution towards calcium-induced mPTP opening is not well established. Reduction of polyP in mitochondria increase a threshold of PTP opening but it is absolutely unclear if it connected to the ion conductivity or direct action on PTP.

Interestingly, another possible component of the bacterial ion channel complex – PHB shown to be involved in mitochondrial calcium uptake, possessing Ca^2+^ ionophoretic activity (Elustondo et al., 2013; Smithen et al., 2013). This raises the question about ability of polyP to modify conductivity of the biological membranes for ions.

Here we used isolated mitochondria to investigate the role of the polyP in mitochondrial ion transport and in activation of PTP. We used the micromolar range of polyP concentrations which was shown for brain (~50 μM) and liver (~100 μM depending on the age) (Lorenz et al., 1997).

## Experimental – materials and methods

### Isolation of mitochondria

Mitochondria were isolated from the liver of Wistar rats (200–250 g) by differential centrifugation. Liver from one rat was homogenized using Teflon-glass homogenizer and resuspended in 50 ml of isolation buffer, which contained 300 mM sucrose, 2 mM EDTA, and 5 mM Tris- HCl, 0.5 mg/ml BSA (bovine serum albumin) pH 7.4. Nuclei and whole cells were centrifuged at 2000×g for 10 min. The supernatant was collected and spun at 6000×g for 20 min. The resulting pellet was re-suspended in 30 ml of the isolation buffer without EDTA and BSA and spun at 7500×g for 20 min. The resulting pellet was re-suspended in 0.5 ml of the isolation buffer without EDTA and BSA and put on the ice-bath.

### Measurements of mitochondrial swelling in isosmotic solutions

The passive permeability of mitochondrial membranes for ions was measured by following energy-independent swelling in isosmotic nitrate solutions as described (Abramov et al., 2001; Brierley, 1974). According to this method, the permeability of mitochondrial membranes can be determined quantitatively and rather simply, based on the kinetics of their energy-independent swelling in various saline solutions. In all investigations of charged particle transport through the inner membrane of mitochondria, the electrical phenomena accompanying these processes should be taken into account. Osmotic swelling in the presence of electrolytes occurs only when both an anion and a cation permeate into the matrix compartment of mitochondria, increasing the osmotic pressure inside the organelle without creating a significant diffusion potential. Application of compounds which induce membrane permeability with known properties and systematic variation of cationic and anionic constituents of the medium permit one to study the permeability of inner mitochondrial membranes for specific ions under normal and experimental conditions.

Nitrate salts of different cations were used to study the passive permeability of inner membranes of mitochondria to cations in the presence of inorganic polyphosphates. Salt concentrations were 120 mM for Na^+^ and K^+^, 80 mM for Mg^2+^, 40 mM for Ba^2+^ and Ca^2+^. In addition, Ca^2+^ and Ba^2+^ solutions contained 120 mM sucrose. All solutions were buffered with Tris-NO_3_ to pH 7.4. To exclude possible energy-dependent transport in these experiments, the incubation medium was always supplemented with rotenone (1 μM). Measurements were performed at room temperature in 2 ml plastic cuvettes. The final concentration of mitochondria, evaluated in terms of protein concentration, was about 0.5 mg/ml. A silicone stirrer was used to continuously mix the recording solution during light scattering measurements.

Stock solutions of polyP standards (sodium salt with polyP content of 60 % (provided by Dr. T. Shiba Regenetiss, Inc., Japan)) were prepared in water to final concentrations of 0.5 mg/ml or 50.

For experiments with energized mitochondria we used following buffers:

1. Tris HCl 10 mM; KCl 120 mM; EGТА 1 mM; glutamate 5 mM; malate 1 mM; KH_2_PO_4_ 2,5 mM.
2. Tris HCl 5 mM; Sucrose 70 mM; Manitol 120 mM; glutamate 5 mM; malate 1 mM; KH_2_PO_4_ 0.2 mM (in some experiments 1 mM EGTA was added in this buffer as well).

Complete mitochondrial swelling in some experiments was achieved by the addition of 5 μM of alamethicin (ala).

### Statistical analysis

Statistical analysis and exponential curve fitting were performed using Origin 8.5 software (Microcal Software Inc., Northampton, MA). Results were expressed as mean ± S.E.

### Planar lipid bilayer experiments

Black lipid bilayer (BLM) experiments were performed as previously described. Briefly, BLM was formed from a 20 mg/ml lipid solution of either DOPC or DOPC:Cardiolipin (3:1) in n-decane (Aldrich). The lipid solution was painted across the 200 μm aperture of a Delrin cup (Warner Instruments, Hamden, CT) to form the planar bilayer. The recording solution contained 150 mM NaCl, (symmetric), 2 mM CaCl_2_ (in cis compartment)/10 mM CaCl_2_ (in trans compartment), 10 mM Tris-HCl, pH 7.4. Currents across the bilayer were recorded using Planar Lipid Bilayer Workstation (Warner Instruments). The cis compartment was connected to the head stage input and the trans compartment was held at virtual ground via a pair of matched Ag/AgCl electrodes. Signals from voltage-clamped BLM were high-pass-filtered at 2.1 kHz using an eight-pole Bessel filter LPF-8 (Warner Instruments), digitized using Data Translation Digitizer and recorded on PC using in-house software developed by Elena Pavlova.

### Measurement of mitochondrial membrane potential

For measurements of ΔΨ_m_, isolated mitochondria were plated on 22 mm glass coverslips and loaded for 30 min at room temperature with 50 nM tetramethylrhodamine methylester (TMRM; Invitrogen) in a medium containing: Tris HCl 10 mM; KCl 120 mM; EGТА 1 mM; glutamate 5 mM; malate 1 mM; KH_2_PO_4_ 2.5 mM. Before imaging the media was replaced with 40 mM Ca(NO_3_)_2_ solution. The dye remained present in the media at the time of recording. Confocal images were obtained using a Zeiss 710 VIS CLSM equipped with a META detection system and a 40× oil immersion objective. TMRM was excited using the 560 nm laser line and fluorescence was measured above 580 nm. For analysis of response to mitochondrial toxins, images were recorded continuously from a single focal plane.

## Results

### PolyP does not change the conductivity of artificial lipid membranes but induces permeability of the native mitochondrial membranes

**Fig. 1:**
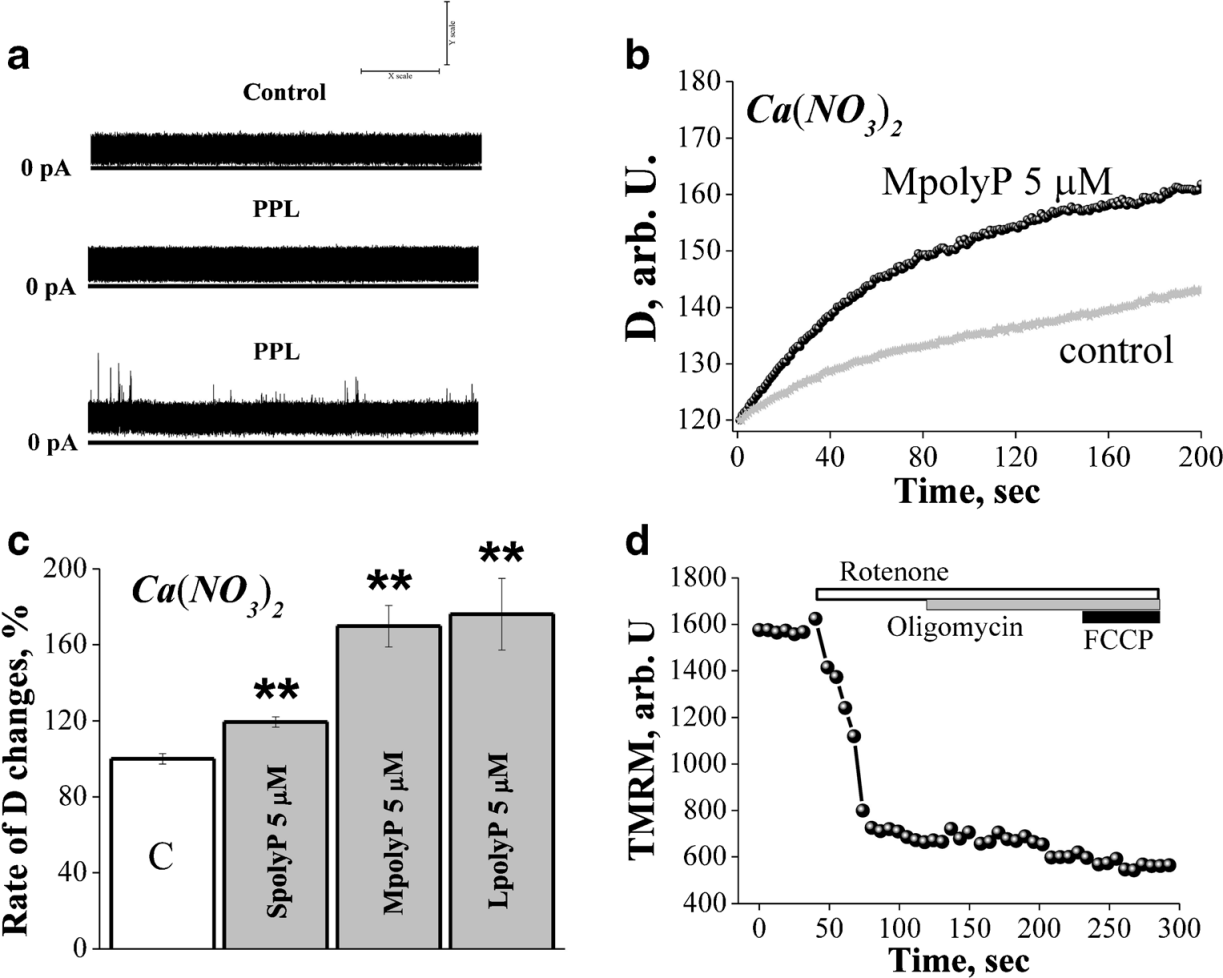
PolyP induces mitochondrial swelling in de-energized mitochondria. **a** PolyP does not change the conductivity of artificial lipid membranes **(n=12)**; **b**Swelling of mitochondria in Ca(NO_3_)_2_ in the presence of 5 μM MpolyP (shown is a representative trace from six independent experiments); D is optical density; **c** Effect of different types of polyP on mitochondrial swelling in Ca(NO_3_)_2_, n=6, difference between the effects of MpolyP and LpolyP is not significant;. **d** rotenone induce complete depolarization of isolated mitochondria in Ca(NO_3_)_2_ solution. 2 μg/ml oligomicin of 1 μM FCCP did not significantly change TMRM fluorescence after application of rotenone. **P≤0.01

Addition of 5–100 μM polyP (short, medium or long) in the experimental chamber was insufficient to increase the conductance of the artificial lipid bilayer (Fig. 1a). In 12 experiments no change in the conductance comparing to control was observed. In 3 experiments we observed brief period of irregular spiking activity, which did not lead to the opening/formation of stable ion channel. Thus, inorganic polyP cannot significantly modify conductance of the artificial lipid membranes for monovalent or divalent cations.

In order to investigate the ability of polyP to stimulate the conductance of the natural mitochondria membranes we used isolated de-energised mitochondria. Mitochondrial ionic transport can be detected from a time dependent swelling of the de-energised mitochondria in isosmotic solutions of the nitrates of divalent and monovalent cations.

We found that application of the short (SpolyP), medium (MpolyP) or long (LpolyP) length polyP (5 μM) significantly increased the rate and amplitude of mitochondrial swelling in Ca(NO_3_)_2_ solutions (Fig. 1b). However, application of the SpolyP led to increase of mitochondrial swelling by 119.4 ± 2.73 %, comparing to control (*n* = 6). Use of polyP of longer chain length further increased its ability to induce swelling. Addition of 5 μM of MpolyP increased mitochondrial swelling to 169.77 ± 10.94 %, (*n* = 6) comparing to control, while addition of LpolyP increased swelling by 176.1 ± 18.91 % (Fig. 1c, n=6).

In order to identify if 1 μM rotenone is enough to induce mitochondrial depolarisation in iso-osmotic solutions we used TMRM as a fluorescent indicator of ΔΨ_m_ in isolated mitochondria (Fig. 1d). Application of rotenone (1 μM) induced profound depolarisation of mitochondria under these conditions. Application of oligomycin (2 μg/ml; Fig. 1d), an inhibitor of the F_1_F_0_-ATPase, induced no response suggesting that the ATP synthase/−ase had no effect of mitochondrial membrane potential in Ca(NO_3_)_2_ solution. Mitochondrial uncoupler FCCP (1 μM) had no effect on TMRM fluorescence suggesting that 1 μM rotenone induce complete mitochondrial depolarisation in Ca(NO_3_)_2_ solution (Fig. 1d).

### PolyP induces a selective transport for Ca^2+^ and Ba^2+^ to mitochondria in a dose-dependent manner

**Fig. 2:**
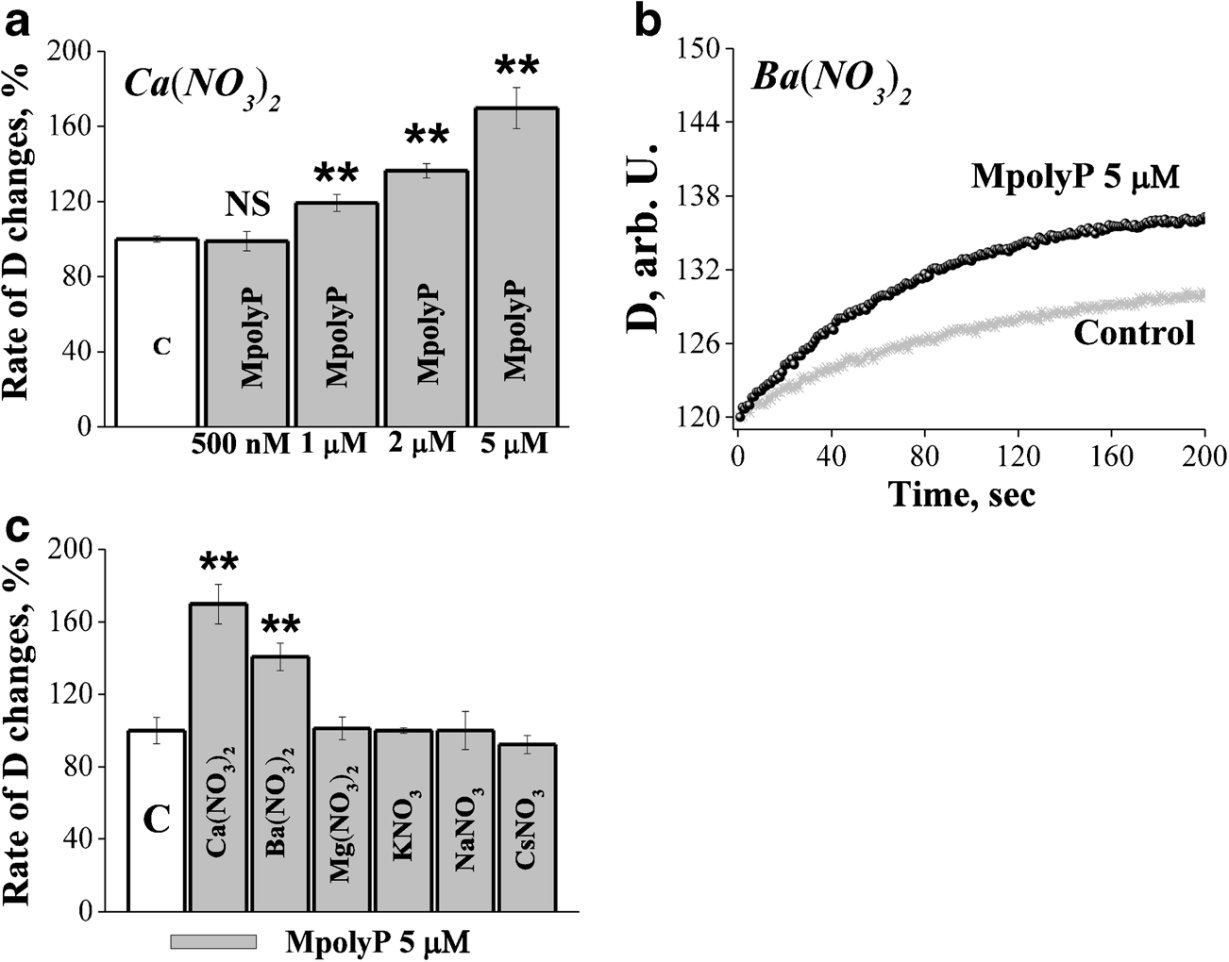
PolyP induces a selective transport for Ca^2+^ and Ba^2+^ to mitochondria in a dose-dependent manner **a** Dose dependence of MpolyP, n=6 for 5 μM; n=9 for 2 μM; n=6 for 1 μM; n=6 for 500 nM; **b** Effect of polyP on mitochondrial swelling in Ba(NO_3_)_2_; **c** Selectivity of polyP dependent mitochondrial swelling for different cations –controls are estimated as 100 % for Ca(NO_3_)_2_. n=6; for Ba(NO_3_)_2_ n=11; for Mg(NO_3_)_2_ n=5; for KNO_3_ n=6; for NaNO_3_ n=6; for CsNO_3_ n=3; **P≤0.01

The effect of the polyP was dose-dependent with the lowest active concentration 1 μM for both M polyP and LpolyP (Fig. 2a). Lower concentrations (500 nM) did not cause any detectable changes in mitochondrial swelling (n=6, p<0.01, Fig. 2a). The lowest active concentration for SpolyP was 2.5 μM (n=5; data not shown).

To test the selectivity of the polyP-induced passive ion transport to mitochondria we performed the experiments with different nitrate solutions of divalent and monovalent cations. However, application of 5 μM polyP of any type had no effect on mitochondrial swelling in solutions of monovalent cations, nor in Mg(NO_3_)_2_ but this effect appeared again in the presence of barium ions (Ba(NO_3_)_2_). Addition of 5 μM of M polyP led to increase of permeability of mitochondrial membrane to 140.72 ± 7.6 %, n=11 (Fig. 2b, c).

Considering the selectivity of polyP to Ca^2+^ and Ba^2+^ and the absence of the effects of the polymer on the artificial membranes, we suggested that effects of polyP can be linked to its ability to modify or activate mitochondrial endogenous calcium transporting systems.

**Fig. 3:**
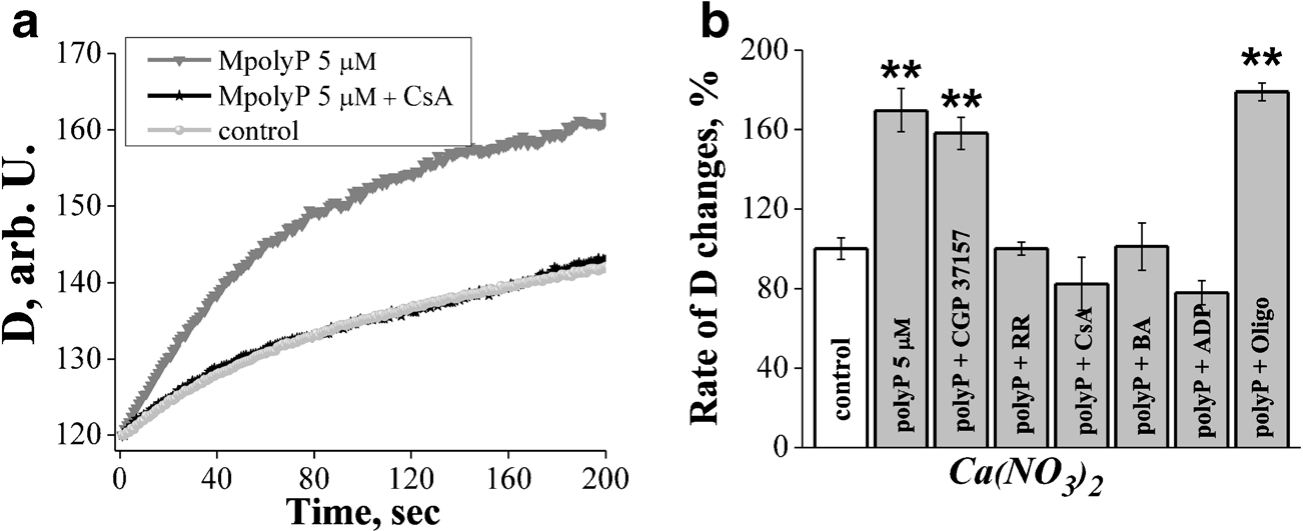
The nature of polyP-dependent swelling. **a** In the presence of 1 μM CsA polyP did not cause increase in mitochondrial swelling in Ca(NO_3_)_2_; **b** Cyclosporine A (0.5 μM n=4, bongkrekic acid (1 μM) n=3, ruthenium red (5 μM) n=6 and ADP (400 μM) n=6 blocked effect of polyP whereas inhibitor of Na^+^/Ca^2+^ exchanger CGP 37157 (10 μM) n=6 and oligomycin (5 μM) n=7 had no effect, **P≤0.01

To identify the pathway through which polyP can stimulate mitochondrial swelling in Ca(NO_3_)_2_ we applied several inhibitors of different mitochondrial transporting systems. Inhibitor of Na^+^/Ca^2+^ exchanger CGP 37157 (10 μM, n=6) did not cause any changes in polyP-dependent swelling, but effect of polyP was completely blocked by pre-incubation of mitochondria with the inhibitor of PTP – cyclosporine A (CsA) in higher concentration (1 μM; Fig. 3a; n=6, p<0.01) or in lower 0.5 μM (Fig. 3b; n=4, p<0.01). To further confirm the involvement of PTP, we performed experiments with its two other inhibitors: bongkrekic acid (inhibitor of adenine nucleotide translocase – ANT (1 μM, n=3) and ADP (400 μM, n=6). Both compounds completely blocked the polyP-induced swelling of mitochondria in Ca(NO_3_)_2_ (Fig. 3b)_._ Interestingly, effect of polyP in Ba(NO_3_)_2_ was also dependent on the presence 0.5 μM CsA (n=8; data not shown). To test the potential contribution of nonspecific transport through mitochondrial ATPase, we performed experiments in the presence of ATPase inhibitor oligomycin (5 μM, n=7) and found that this compound do not alter polyP- induced mitochondrial swelling in Ca(NO_3_)_2_ (Fig. 3b). Interestingly, inhibitor of mitochondrial calcium uniporter Ruthenium Red (5 μM, n=6) also completely inhibited effect of polyP (Fig. 3b). This result is surprising considering the electrogenic nature of uniporter and de-energized mitochondria, which had been used for experiments. This effect may be explained by the direct effect of polyP on mitochondrial calcium uniporter or any other unspecific effect of Ruthenium Red on mitochondrial transporters.

Thus, polyP induces mitochondrial swelling via opening PTP. This effect can be secondary to activation of the mitochondrial calcium uniporter and Ca^2+^, which lead to PTP opening and swelling of mitochondria. In order to identify the target of polyP (calcium uniporter or PTP) we used intact mitochondria.

### PolyP modulates calcium dependent permeability transition pore in lower concentrations and causes calcium independent swelling in energized conditions

**Fig. 4:**
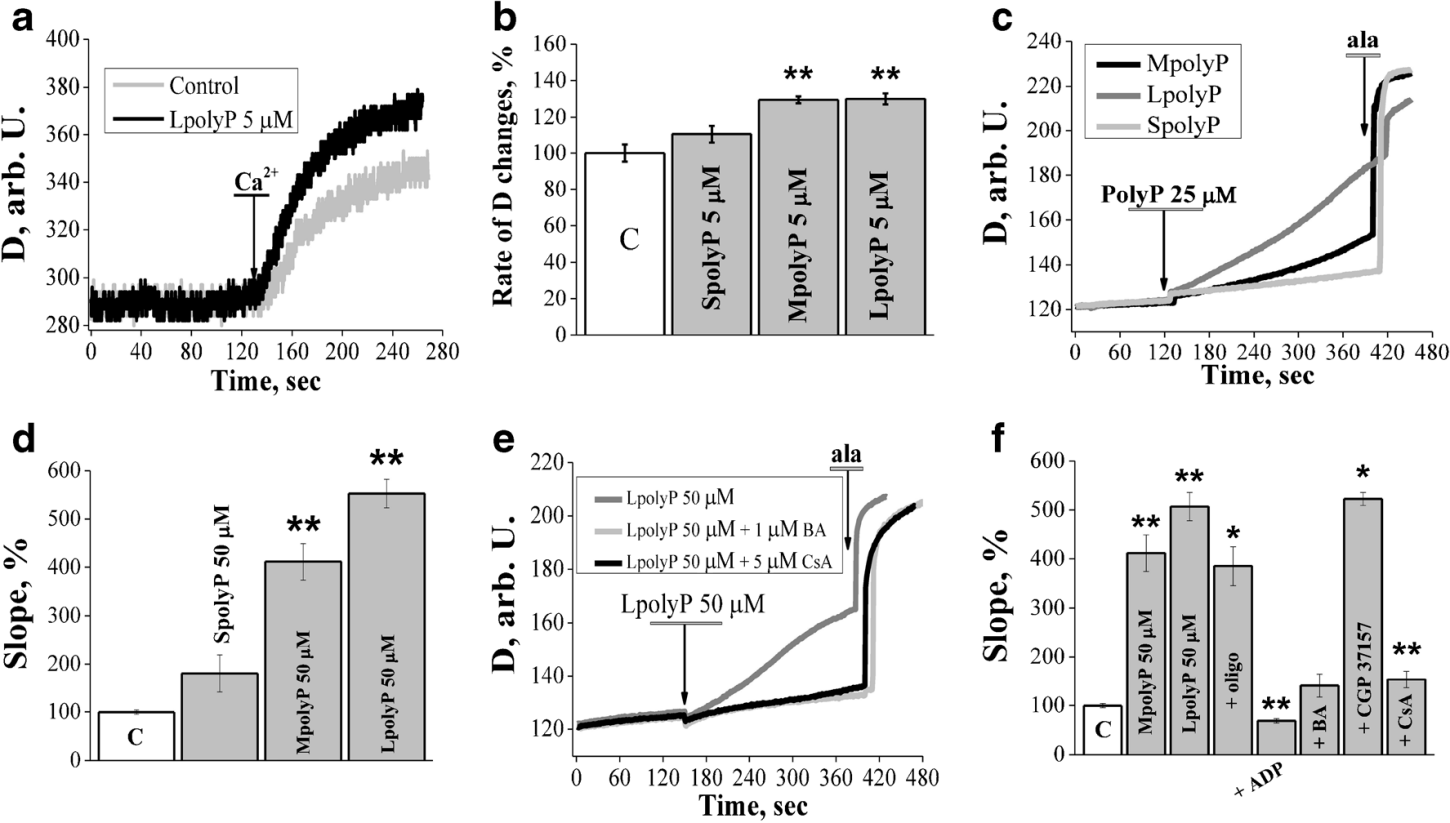
PolyP modulates calcium dependent permeability transition pore in lower concentrations and causes calcium independent swelling in energized conditions. **a, b** LpolyP and MpolyP in concentration 5 μM increased the amplitude of Ca^2+^ induced swelling (n=6); **c** higher concentrations of medium and long polyP (25 μM) can trigger mitochondrial swelling by itself without added calcium ions (n=6;6;6 for short medium and long polyP respectively); **d** PolyP in concentration 50 μM caused mitochondrial swelling independently on the length of polyP (n=6; 12; 21 for short medium and long polyP respectively); **e** CsA and BA totally inhibited polyP induced swelling (n=7 for CsA and n=3 for BA); **f** Effect of different inhibitors for polyP induced swelling in energized conditions (n=6 for oligomycin, n=7 for ADP, n=3 for BA; n=6 for CGP37157 and n=7 for CsA. **P≤0.01

Application of low concentration (5 μM) of short, medium or long polyP did not induce the mitochondrial swelling in energized conditions by itself. However, medium and long polyP increased the amplitude of swelling after induction of PTP by calcium ions (the threshold concentration of calcium which opened PTP varied in the range of 25–50 μM (for mitochondrial concentration 0.5 mg/ml of protein, Fig. 4a, b). It should be noted that short polyP was not as potent as medium and long polymers. Interestingly, higher concentrations of medium and long polyP (25 μM) can trigger mitochondrial swelling by itself without added calcium ions (plus 1 mM EGTA, Fig. 4c). Further increase of polyP concentration to 50 μM caused mitochondrial swelling independently on the length of polyP. Application of 50 μM of SpolyP increased the rate of swelling to 180.34±38.72 % (n=6), MpolyP to 411.5±37.41 % (n=12) and LpolyP to 506.92±29.09 % (n=21) of control (Fig. 4d, p<0.01). The rate of mitochondrial swelling was also dependent on the concentration of polyPs. The lowest concentration of LpolyP, which cased mitochondrial swelling was 12.5 μM, when it increased the rate of mitochondrial swelling to 155.47±21.4 % of control, (data not shown, n=5). For MpolyP the lowest concentration which induced swelling was 25 μM (246.48±21.36 %, n=6).

Consistent with the results obtained using de-energised mitochondria, we found that responses were dependent on PTP inhibitors bongkrekic acid (n=3), ADP (n=7) and CsA (n=7), but independent of CGP 37157 (n=6) and oligomycin (n=6) (Fig. 4e and f, p<0.01). Interestingly, Ca^2+^ and polyP-induced PTP swelling of mitochondria could be blocked by 1 μM CsA, while block of swelling induced by polyP only required higher concentration of CsA (5 μM; n=7).

## Discussion

Here we show that external polyP can trigger PTP opening. Short or long polymer can enhance the effect of calcium, but very importantly, can stimulate pore opening without calcium. Previously, we and others shown that changes of polyP levels inside mitochondria can modulate PTP and that, in combination with PHB polyP can form channel with properties resembling PTP (Abramov et al., 2007; Pavlov et al., 2005; Seidlmayer et al., 2012). Ability of polyP to activate PTP without calcium raises the possibility that calcium might be required as a factor that decreases the threshold of polyP activation of PTP rather than plays the role of the essential co-factor.

Phosphate is known to be an important regulator of the PTP (Basso et al., 2008; Crompton et al., 1988; Li et al., 2012). However, the effects of polyP cannot be explained by simple increase of the phosphate concentration. In our experiments SpolyP had much smaller effects than longer polymers suggesting the importance of the polymer size rather than total concentration of phosphate groups.

Although it is established, that decrease in mitochondrial membrane potential can trigger PTP (Bernardi, 1992), in our experiment we found that PTP can be open in de-energised mitochondria when mitochondria are expected to be completely depolarised. Interestingly, under these conditions ADP and bongkrekic acid were able to block this potential independent PTP opening, suggesting the involvement of the Adenine Nucleotide Translocase (ANT) and/or ATPase.

Previously we found that production of the polyP in mitochondria is dependent on mitochondrial potential and can be block by oligomycin, suggesting the role of F_0_-F_1_-ATPsyntase in polyP synthesis (Angelova et al., 2014; Pavlov et al., 2010). This suggests that ATPase is a protein capable of interacting with polyP. Considering the recent finding the role F_0_-F_1_-ATPsyntase in PTP formation (Giorgio et al., 2013; Halestrap, 2014) it is tantalizing to suggest that long chain polyP may be a trigger or bridge for dimerization of complex by binding phosphate binding sites of two F_0_-F_1_-ATPsyntase by one long molecule of polyP. These interactions might not require the presence of the increased amounts of calcium. In the light of this inhibitory effect of ADP even in the conditions of absence of mitochondrial membrane potential can be induced by higher affinity of ADP to F_0_-F_1_-ATPsyntase. We should note that in our experiments order to affect ATPase polyP would be expected to cross the mitochondrial outer membrane, presumably through VDAC channel. However, taking into account the non-essential role of VDAC in PTP it is very unlikely that modulation of this channel function by polyP plays dominant role in the observed effects but can be important for transport of polyP into mitochondria.

In summary, our data show that externally added polyP can stimulate mitochondrial swelling in de-energized (for Ca^2+^ and Ba^2+^ ions) and energized conditions, which was dependent on classical inhibitors of PTP. The results presented in this study demonstrate that polyP in the concentrations 25 and 50 μM, which are physiological for mammalian tissues (Gabel and Thomas, 1971; Kumble and Kornberg, 1995), can stimulate opening of the PTP without the addition of external calcium. Considering the ability of polyP to induce PTP opening by itself we can suggest important role in regulation of the signal of the cell death by long polymer that in agreement with function of polyP as primordial molecule (Gray et al., 2014).
